# Health Risk and Biological Effects of Cardiac Ionising Imaging: From Epidemiology to Genes

**DOI:** 10.3390/ijerph6061882

**Published:** 2009-06-19

**Authors:** Ilenia Foffa, Monica Cresci, Maria Grazia Andreassi

**Affiliations:** 1 Institute of Clinical Physiology, CNR, Pisa, Italy; E-Mails: ilenia@ifc.cnr.it (I.F.); cresci@yahoo.it (M.C.); 2 Genetic Research Unit, G. Pasquinucci Hospital, Via Aurelia Sud-Montepepe, 54100 Massa, Italy

**Keywords:** ionizing radiation, DNA damage, cancer risk, biomarkers, genetic polymorphisms

## Abstract

Cardiac diagnostic or therapeutic testing is an essential tool for diagnosis and treatment of cardiovascular disease, but it also involves considerable exposure to ionizing radiation. Every exposure produces a corresponding increase in cancer risk, and risks are highest for radiation exposure during infancy and adolescence. Recent studies on chromosomal biomarkers corroborate the current radioprotection assumption showing that even modest radiation load due to cardiac catheter-based fluoroscopic procedures can damage the DNA of the cell. In this article, we review the biological and clinical risks of cardiac imaging employing ionizing radiation. We also discuss the perspectives offered by the use of molecular biomarkers in order to better assess the long-term development of health effects.

## 1. Introduction

The increasing exposure to medical radiation in Western countries is a hot issue that the medical community needs to appreciate because it may likely result in an increase in the incidence of imagingrelated cancer in the future [1–3].

Medical use of radiation is the largest man-made source of radiation exposure to the general population [1]. According to the recent report on medical radiation exposures to the population in the United States, the *pro-capite* collective dose of radiation received from clinical imaging has increased by > 700% between 1980 and 2006 [2].

The current annual collective dose radiation exposure received by the US population has been calculated as roughly equivalent to the total worldwide collective dose generated by the nuclear catastrophe at Chernobyl [2,3].

Many cardiac diagnostic or therapeutic testing, such as cardiac catheterization, CT and nuclear medicine scans, accounting for > 50% of all imaging examinations, involve considerable exposure to ionizing radiation [4–6]. A contemporary cardiac patient is exposed to a significant cumulative effective dose (a median cumulative effective dose of 60 mSv per head) from multiple tests, often repeated needlessly [6,7].

Three central principles provide the foundation for radiation protection: 1) justification (the benefit of radiation exposure outweighs any accompanying risk; 2) optimization (total exposure remains as low as reasonably achievable, ALARA principle), and 3) dose limits (dose limits are applied in order to ensure that no one is exposed to an unacceptably high risk).

In cardiological practice, therefore, every effort should be done to justify the indications and to optimize the doses during ionising testing. The imaging examination should confer a relevant clinical information on patient management, i.e. the benefit deriving from the examination must be greater than the long-term risk associated with the exposure [6,8].

The evaluation of the health effects of low-dose ionizing radiation has always been the main issue in radiological protection. In this article, we review the biological and clinical risks of diagnostic imaging employing ionizing radiation as well as to discuss the perspectives offered by the use of surrogate molecular biomarkers in order to better assess the long-term development of health effects.

## 2. Biological Effects of Ionizing Radiation

The biological effects of ionizing radiation are divided into two categories: deterministic and stochastic effects [9–13]. Deterministic effects, such as *erythema* or *cataracts*, have a threshold dose below which the biological response is not observed (Table 1). Some cardiological interventional procedures with long screening times and multiple image acquisition (e.g. percutaneous coronary intervention, radio-frequency ablation, etc.) may give rise to deterministic effects in both staff and patients [14,15].

A stochastic effect is a probabilistic event and there is no known threshold dose (Table 1). The likelihood of inducing the effect, but not the severity, increases in relation to dose and may differ among individuals. In fact, the effect of low doses of radiation – less than 50 mSv – do not cause an immediate problem to any body organ, but spread out over long periods of time after exposure.

Damage to DNA, which carries the genetic information in chromosomes in the cell nucleus, is considered to be the main initiating event by which radiation damage to cells results in development of cancer and hereditary disease in the future children of exposed parents [9–13].

Ionizing radiation exposure produces long-term health risk through, both directly or indirectly (free radical interaction), damage to cellular DNA, producing oxidized bases, bulky DNA adducts, and DNA strand breaks.

The cell has repair mechanisms against damage induced by radiation as well as by chemical carcinogens. Consequently, biological effects of low dose radiation on living cells may result in three outcomes: (1) injured or damaged cells repair themselves, resulting in no residual damage; (2) cells die; or (3) cells incorrectly repair themselves resulting in a biological change.

**Figure 1.**
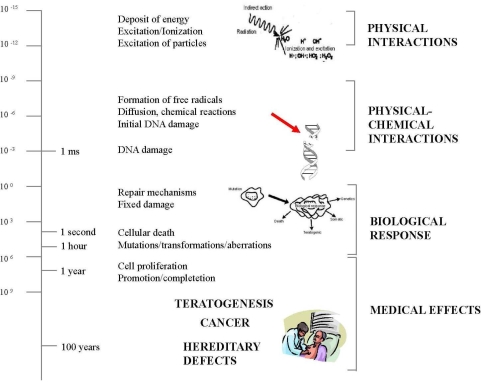
Chemical and biological effects by ionizing radiation. On the left side: from physical interaction (a few milliseconds) to clinical effects (decades later). On the right side: the corresponding molecular (DNA damage), cellular (cell damage or proliferation), and clinical events (such as cancer). Redrawn and modified from ref. [10].

Therefore, the biological effects of a stochastic effect are at DNA level and they may not be detected. The basic concept is that physical steps that lead to energy deposition and free radical formation occur within 10^−5^ to 10^−6^ seconds, whereas the biological expression of physical damage may occur a second or decades later [10] (Figure 1).

Radiation–induced mutation contribute to the multi-step process of human cancer arising from the accumulation of multiple genetic abnormalities (over-expression of genes, deletion of genes, or gene mutations), some of which must occur in critical genes that regulate proliferation and differentiation. Genetic effects are the result of a mutation produced in the reproductive cells of an exposed individual that are passed on to their offspring. These effects may show up as birth defects or other conditions in the future children of the exposed individual and succeeding generation.

Adverse hereditary effects that could be attributed to radiation have not been found, in epidemiological studies of children whose parents were exposed to radiation [12,13]. However, studies conducted on mice and other organisms have produced extensive data showing that radiation-induced cell mutations in sperm and eggs can be passed on the offspring [12]. Thus, there is no reason to believe that humans would be immune to this sort of harm [12].

## 3. Clinical Risk of Medical Ionizing Radiation Exposure

Carcinogenesis, teratogenesis and heritable effects are the main health risks with ionizing radiation. Other non-cancer adverse effects -especially atherosclerotic cardiovascular and cerebrovascular riskcan occur following high-dose radiation therapy, but more research is needed to fully assess these outcomes at low and moderate doses [12,13].

Radiation risks are reviewed at regular intervals by international and national radiological organizations by considering scientific progress worldwide in order to reach a balanced view of the risks involved.

The current consensus of these regulatory bodies is that for radiation protection purposes the most appropriate risk model at low doses is the so-called linear no-threshold (LNT) model, without threshold safe dose.

During last years, however, two opposing concept to LNT model have emerged. In fact, some have argued that risks are smaller than predicted by the linear no-threshold model [16], or that low doses of radiation may even be beneficial because organisms possess the ability to respond to low-dose radiation by stimulating certain protective functions (*radiation adaptive response*), including antioxidative capacity, DNA repair functions, apoptosis [17].

Some postulate that low doses of radiation are more harmful than previously thought because damage occurs not to the cell that was exposed to radiation but also to surrounding cells (*bystander effects*) [18].

The most recent update of the health risks of exposure to low levels of ionizing radiation comes from the National Research Council Committee on the Biological Effects of Ionizing Radiation Report (BEIR VII) of the National Academy of Sciences [12].

The evidence considered by BEIR VII comprises epidemiological studies of human populations, including atomic bomb survivors, patients exposed to radiation from diagnostic and therapeutic medical studies, as well as studies from occupational exposures and from exposure due to releases of radioactive materials into the environment [12,19].

Direct epidemiological evidence (A-bomb survivors and other groups) demonstrated that there is a linear relationship between risk of cancer and dose between about 50 mSv and 2.5 Sv, but the risk of cancer associated with lower dose remains uncertain, because the natural incidence of cancer in any population is high.

BEIR VII still reconfirmed that the LNT model is the best model to estimate radiation risks, continuing to support the well-established radiobiological concept that no radiation doses -no matter how small- can be considered completely safe.

BEIR VII indicated that a single adult population effective dose of 10 mSv results in a 1 in 1,000 lifetime risk of developing radiation-induced solid cancer or leukaemia [12]. However, approximately 42 people out of 100 are expected to develop cancer for other reasons [12].

It is worth noting that many cardiac ionizing procedures have effective dose estimates in the range of 10 to 25 mSv [7]; thus, it is not be uncommon for a patient to exceed the dose of 50 mSv, even in a single hospital admission for a single problem, most commonly a suspicion of coronary artery disease.

**Figure 2.**
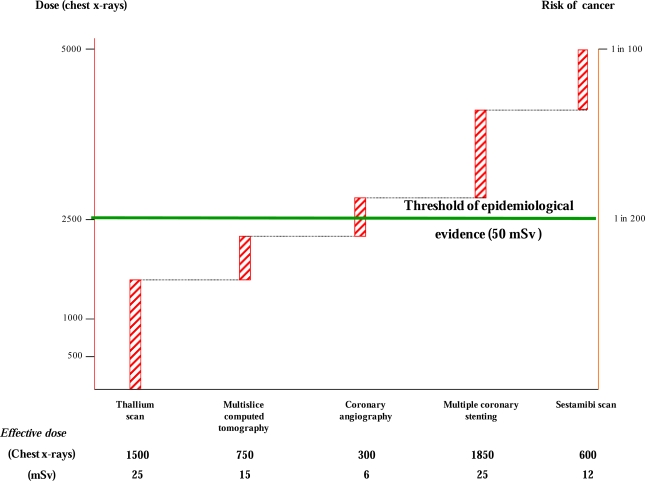
Graphical representation the cumulative exposure of doses in multiples of dose from a simple chest x ray (y axis, left) and corresponding cancer risk (y axis, right) cancer risk and radiation dose (in multiples of dose from a simple chest x ray) for a typical cardiological patients undergoing to five radiological examinations. Redrawn and modified from ref. [4].

For instance, a 50-year-old man who undergoes one thallium scan stress tests, one 64-slice computed tomography coronary angiography, one coronary angiography, and one coronary intervention would receive an effective radiation dose of about 71 mSv and thus would have an additional subsequent lifetime risk for developing cancer of about 1 in 150 patients (Figure 2).

**Figure 3.**
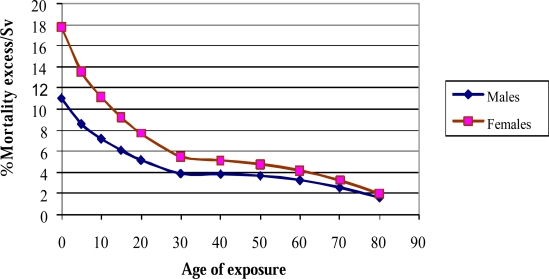
Radiation- Induced Risk of Cancer on Age and Gender by using the BEIR VII estimates.

Furthermore, it is very important to underline those children and young adults are especially vulnerable since they have more rapidly dividing cells and a greater life expectancy [12]. For example, the overall risk of developing cancer (incidence and mortality) from the same dose of radiation for a 1-year old infant is 10–15 times greater than a 50 year old adult, and female infants have almost double the risk than that of male infants. Figure 3 shows the estimated risk of cancer mortality as a function of age at exposure for both males and female.

Therefore, alternative diagnostic modalities that do not involve the use of ionizing radiation should be considered in the evaluation of young individuals in order to minimize cancer risk [20,21].

## 4. The Limitation of Risk Assessment from Epidemiological Studies

Epidemiological studies are meritorious and important in order to estimate the health risk from radiation exposure. However, quantifying the risk at low dose below 50 mSv in humans may not be accurately estimated by any epidemiological studies because of a very high background incidence rate of cancer and numerous confounding factors. Most epidemiological studies have inherent limits of being statistically underpowered because studies with very large sample size are required in order to quantify the risks of very low doses of radiation. For instance, it has been estimated that more than 5 million people are required in order to directly quantify the risk of cancer from exposure to doses of radiation of 10 mSv or less [22].

At low doses, the risk is essentially estimated by extrapolation of the dose-effect curve obtained from high doses. Because of limitations in the data used to develop risk models, risk estimates are uncertain, and estimates that are a factor of two or three larger or smaller cannot be excluded [12].

Therefore, additional research is needed to better understand the biological events that lead to the development of most cancers, as recently indicated in the Recommended Research Needs by the BEIR VII report [12].

In particular, the BEIR VII committee has recommended medical studies of patients, especially infants who have had a significant medical radiation, such as exposure related to cardiac catheterization [12].

More research is also recommended needed on DNA damage chromosomal aberrations, and gene mutations caused by radiation exposure, as well as the role of genetic susceptibility in modulating low dose radiation response [3,12].

In order to overcome the severe practical limitations of the epidemiological approach, an alternative strategy is based on the use of *biomarkers* as early predictors of delayed health outcomes and susceptible populations [23].

## 5. Biomarkers in the Assessment of Radiation Exposure Support BEIR VII Estimates

The incorporation of molecular and cellular biomarkers (molecular epidemiology) into epidemiological studies has grown exponentially during recent years in order to better examine relationships between environmental hazards and human health effects. The use of biomarkers is expected to identify important mechanistic insight into the pathogenesis of disease processes and reduce the time gap exposure and recognition of disease-relevant effects.

**Figure 4.**
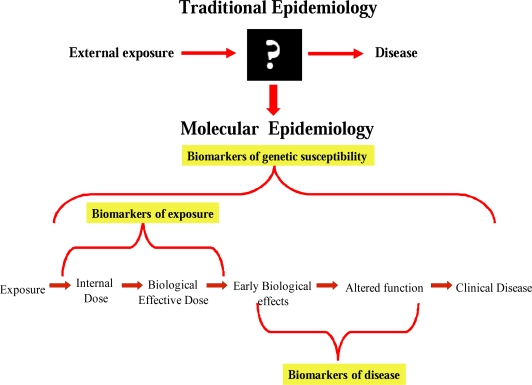
Biomarkers are the key elements of molecular epidemiology and may open the “black box” from exposure to disease.

One of the goals of molecular epidemiology studies is to use biomarkers in order to develop new and more effective strategies to reduce risk, such as exposure monitoring, health surveillance and individual risk characterization [24,25]. Different biomarkers reflecting exposure to and early effects of carcinogens, as well as individual genetic susceptibility to them, have become available and are being applied in population-based (*molecular epidemiology*) (Figure 4).

**Figure 5.**
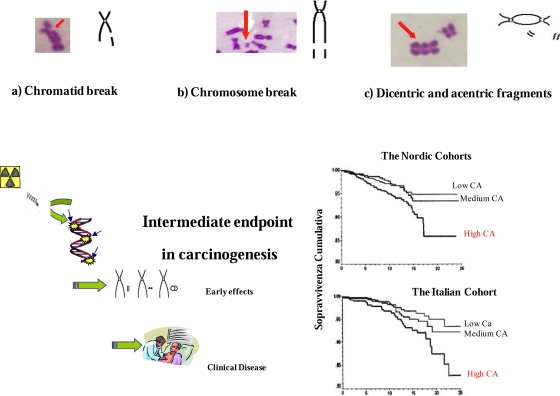
Chromosomal aberrations in peripheral blood lymphocytes: biomarkers of early effect and cancer risk assessment. Redrawn and modified from ref. [26].

Biomarkers of early effect with relevance to the carcinogenic process include the evaluation of chromosomal DNA damage in peripheral blood lymphocytes in the form of chromosome aberrations and micronuclei [23]. During recent years, indeed, large studies have provided consistent evidence that high levels of chromosomal DNA damage in peripheral blood lymphocytes are early predictors of cancer risk [26,27]. The use of chromosomal biomarkers may assist in the difficult task of assessing the risk of radiation-induced oncogenic effects. As such, they can complement classic epidemiological studies that use disease endpoints and require millions of people followed-up for several decades (Figure 5).

Indeed, we have recently used chromosomal biomarkers as intermediate endpoint in carcinogenesis in order to assess the potential risk due to cardiac catheter-based fluoroscopic procedures, magically in the line of Research Needs as outlined by the BEIR VII report on 2006 [12] and by the White Paper of the American College of Radiology on effects of medical radiation released on 2007 [3]. Our results corroborated the current radioprotection assumption that even modest radiation load can damage the DNA of the cell.

Invasive cardiovascular procedures can damage the DNA of the cell to be detectable-acutely and in the long-term as increased chromosomal DNA damage in circulating lymphocytes that represent an intermediate endpoint of cancer [28,29].

Importantly, we observed that the lifetime exposure of a young adolescent with congenital heart disease in the range of 20 mSv is associated with a dramatically 200% increased frequency of chromosomal DNA damage when compared to age- and sex-matched control subjects [28]. Furthermore, we also showed that contemporary interventional cardiologists have an increased rate of chromosomal somatic DNA damage, reflected in higher frequency of micronuclei versus clinical cardiologists [30].

Actually, interventional cardiologists have a *per-capita* per year exposure two-to three times higher than that of radiologists [31]. Cumulative doses after 30 years of working life can be as high as 100 to 250 mSv, corresponding to a whole body dose equivalent to 5,000 to 12,500 chest X-rays. This exposure gives an estimated lifetime attributable risk of cancer incidence in the range of 1 cancer in 100–1 in 200 exposed subjects [32].

However, the amount of this damage varies and is only weakly related to the duration of professional exposure, suggesting that an individual predisposition may play an important role in the cellular response to radiation exposure.

Indeed, it is believed that genetic factors may play a crucial role in cellular responses to radiation and those common single-nucleotide polymorphisms (SNPs) in DNA repair genes can lead to heritable predisposition to cancer. Accordingly, considerable effort is being expended in the search for SNPs involved in different DNA repair pathways, including base excision repair (BER) pathway and double strand break repair process (DSB), which might act as cancer susceptibility genes [33–35].

Interestingly, we recently found that harbouring two or more risk alleles of DNA repair genes, contribute to chromosomal DNA damage levels in interventional cardiologists, suggesting that the risk estimates at the population level can be highly inaccurate at the individual level [36].

A significant association between breast cancer risk and genetic polymorphisms has been also reported among women exposed to low levels of ionizing radiation from medical procedures [37,38]. Subgroups of women who are carriers of mutation in DNA repair genes including BRCA-1 and BRCA-2, showed a significantly increased breast cancer risk associated with exposure to diagnosticXrays, especially to the chest [37,38].

Therefore, the application of biomarkers in molecular-epidemiological researches constitutes a promising new strategy for enhancing exposure assessment as well as for a better understanding of the mechanisms of action and dose-response relationships for ionizing radiation and human cancer.

In general, there is a need to continue epidemiological as well as to integrate these investigations with laboratory studies in order to provide new insights in low dose radiation risks, particularly encountered in modern medicine.

Future molecular epidemiologic studies incorporating genetic polymorphisms and biomarkers of early effects provide the rationale for identifying at-risk susceptible or resistant subpopulations in order to develop better radiation protection programmes.

## Figures and Tables

**Table 1.** Biological effects of ionizing radiation.

	**Deterministic effects**	**Stochastic effects**

**Dose**	Medium-High	Low
**Occurrence time**	Short	Long
**Threshold dose**	Yes	No
**Cell Biology**	Cell Death	DNA damage
**Clinical effects**	Skin lesions, erythema, ulcers, epilation, cataracts, permanent sterility	Cancer, genetic effects
